# Neutrophil Reverse Migration Becomes Transparent with Zebrafish

**DOI:** 10.1155/2012/398640

**Published:** 2012-07-12

**Authors:** Taylor W. Starnes, Anna Huttenlocher

**Affiliations:** ^1^Microbiology Doctoral Training Program and Medical Scientist Training Program, University of Wisconsin-Madison, Madison, WI 53706, USA; ^2^Department of Pediatrics and Department of Medical Microbiology and Immunology, University of Wisconsin-Madison, Madison, WI 53706, USA

## Abstract

The precise control of neutrophil-mediated inflammation is critical for both host defense and the prevention of immunopathology. In vivo imaging studies in zebrafish, and more recently in mice, have made the novel observation that neutrophils leave a site of inflammation through a process called neutrophil reverse migration. The application of advanced imaging techniques to the genetically tractable, optically transparent zebrafish larvae was critical for these advances. Still, the mechanisms underlying neutrophil reverse migration and its effects on the resolution or priming of immune responses remain unclear. Here, we review the current knowledge of neutrophil reverse migration, its potential roles in host immunity, and the live imaging tools that make zebrafish a valuable model for increasing our knowledge of neutrophil behavior in vivo.

## 1. Introduction

“Certain of the lower animals, transparent enough to be observed alive, clearly show in their midst a host of small cells with moving extensions. In these animals the smallest lesion brings an accumulation of these elements at the point of damage. In small transparent larvae, it can easily be shown that the moving cells, reunited at the damage point do often close over foreign bodies [1].” Ilya Mechnikov, one of the fathers of immunology, spoke these words at his Nobel Prize lecture in 1908. More than one hundred years after his seminal studies using transparent starfish larvae to illuminate a role for phagocytosis in immunity, we are again exploiting the power of transparent larvae for research on the immune system. Studies of neutrophils in both humans and mammalian model systems have brought great advances in our knowledge of their functions; however, zebrafish, a small tropical fish with transparent larvae, have demonstrated that direct observation of neutrophils in live animals can provide important insights that would have otherwise faced significant technical challenges using mice.

Neutrophils are the most abundant leukocytes in both humans and zebrafish, and they are critical for defending the host against microbial infection [2]. In response to wounding, infection, or other inflammatory stimuli, neutrophils are rapidly recruited to perform their well-known effector functions: degranulation, phagocytosis, production of reactive oxygen species (ROS), secretion of proinflammatory cytokines, and extrusion of neutrophil extracellular traps (NETs) [3, 4]. These responses are acknowledged to kill and sequester microorganisms at their site of entry and promote the activation of the adaptive immune system [4]. Until recently, it was thought that neutrophils, which responded to a wound, had a single fate: death [5, 6]. There remains clear evidence for neutrophil apoptosis in the abundance of pus that emanates from infected wounds, and the clearance of dead neutrophils from the site of inflammation has been demonstrated to occur through phagocytosis by macrophages [4–7]. However, studies of neutrophil wound responses using live zebrafish embryos revealed for the first time that neutrophils could leave an extravascular site of inflammation and persist in the host [8, 9]. This reverse migration process requires two distinct, but related steps: migration of neutrophils away from the inflamed area and reverse transendothelial migration to enter the vascular lumen.

The observation that neutrophils can reverse migrate away from a wound in zebrafish [8] and in mice [10] raises new questions about neutrophil functions. First, the mechanism that neutrophils use to perform reverse migration and the signals that trigger it are entirely unknown. The fate of reverse-migrated neutrophils also remains to be explored. However, recent studies have demonstrated that neutrophils can affect the adaptive immune system and regulate systemic inflammation in previously unappreciated ways, raising intriguing possibilities for the roles of reverse-migrated neutrophils. Because of the conservation of functions between human and zebrafish neutrophils, as well as the many tools available for live imaging and genetic manipulation, zebrafish will certainly continue to be a critical resource for elucidating the mechanisms and functions of neutrophil reverse migration. Here, we review the features of zebrafish and some of the tools that make them particularly well suited to this task. Additionally, we will discuss the current state of the neutrophil reverse migration field and its implications for the regulation immune responses.

## 2. Zebrafish as a Model for Studies of Immunity

An important feature of any model organism is the ability to infer similarity of function with the species of interest, typically humans. The high conservation of immune cell lineages and effector functions indicates the suitability of zebrafish as a model through which we can better understand the human immune system. Zebrafish have many immune cell lineages in common with humans: neutrophils [8, 11, 12], macrophages [11, 13–15], T cells [16], B cells [17], mast cells [18], eosinophils [11, 19], and basophils [11]. However, the 2–4 days-post-fertilization larvae used for most zebrafish neutrophil research do not have T or B cells [16]. Particularly important for the study of neutrophil reverse migration is the conservation of function within the innate immune system. Like human neutrophils, zebrafish neutrophils are the first responders to inflammatory stimuli, where they are able to phagocytose bacteria and degranulate [15, 20, 21]. Further support for the conservation of neutrophil functions is in the recapitulation of neutrophil phenotypes in zebrafish models of Wiskott-Aldrich syndrome (WAS), warts-hypogammaglobulinemia-infections-myelokathexis (WHIM) syndrome, and leukocyte adhesion deficiency-(LAD-) like syndrome [22–24]. Many other immune effector functions are present in both fish and mammals, and these have been expertly reviewed elsewhere [25–28].

The genetic tractability of zebrafish is another attractive point of this model system, and the tools available for genetic manipulation are rapidly improving. Historically, suppression of gene expression in zebrafish has been performed by morpholino oligonucleotides, nucleic acid analogs that bind pre-mRNA to prevent splicing or the initiation of translation [29]. This method has the disadvantage of being transient and affecting the entire organism. However, more recent developments indicate that shRNA-mediated knockdowns are possible in zebrafish, which should allow the creation of transgenics with tissue-specific knockdowns [30]. While previous knockout technology relied on random mutagenesis and screening, new approaches promise to increase the availability of knockout zebrafish to the community [23, 31]. Zinc finger nucleases (ZFN) and transcription activator-like effector nucleases (TALEN) both rely on modular DNA recognition motifs coupled to nucleases to introduce highly localized DNA lesions [32–34]. Tissue specific, inducible gene expression systems, such as those that rely on cre-mediated recombination, are actively being developed and will be critical in assessing the functions of genes whose long-term expression is detrimental [35–37]. The relative ease of transgenesis and the growing complement of tools for genetic manipulation are a very attractive point of the zebrafish model and should drive advances in understanding leukocyte behavior.

Perhaps the single most significant advantage of zebrafish larvae as a model organism is their optical clarity, which allows for noninvasive, live imaging. Furthermore, live zebrafish imaging can be accomplished with commonly available confocal microscopes or fluorescence stereomicroscopes, obviating the need for highly specialized equipment and techniques used for in vivo imaging in mice. The ability to perform live imaging in zebrafish has been enabled by the discovery of cell lineage-specific promoters that can drive expression of fluorescent proteins that label cells or other proteins of interest. Two promoters, myeloperoxidase (mpx) and lysozyme c (lyz), are used to drive neutrophil-specific expression [8, 12, 38, 39]. Recent advances have also allowed tissue-specific expression in macrophages using the macrophage-expressed gene-1 (mpeg1) and colony stimulating factor 1 receptor (csf1r) promoters [14, 15]. These promoters have enabled the creation of stable transgenic lines and the characterization of neutrophil or macrophage responses to wounds, infections, and other inflammatory stimuli.

## 3. Imaging Tools Used to Advance Zebrafish Research

The use of tissue-specific expression with powerful imaging tools has facilitated the application of a cell biology toolkit to zebrafish inflammation research and increased our fundamental knowledge of neutrophil motility and wound recruitment. The first studies utilizing fluorescent neutrophils in zebrafish larvae demonstrated that neutrophils rapidly respond to mechanical wound-induced stimuli, which raised two fundamental questions: (1) What are the intracellular signals that promote directional migration and (2) What are the signals recruiting neutrophils to wounds? Advances in zebrafish imaging strategies have helped to answer both of these questions.

In order to query the signaling responsible for neutrophil motility and wound responses, Yoo et al. applied several imaging techniques from cell biology [40]. The GFP-tagged ratiometric probe, pleckstrin homology domain of Akt (PHAKT-EGFP), allowed live imaging of PI(3,4)P_2_-PI(3,4,5)P_3_ inside of neutrophils. Additionally, this study made use of photoactivatable Rac, whose activity could be induced in individual neutrophils with the targeted application of 458 nm laser light [41]. Finally, the F-actin probes, Lifeact and utrophin calponin homology domain (UtrCH), allowed simultaneous in vivo imaging of total F-actin and stable F-actin, respectively [42, 43]. Imaging of PHAKT-GFP demonstrated that PI(3,4)P_2_-PI(3,4,5)P_3_ accumulated at the leading edge of migrating neutrophils, and inhibition PI(3)K prevented leading edge PI(3,4,5)P_3_ production, leading edge protrusion, and neutrophil motility. While photoactivation of Rac in normal neutrophils could be used to precisely control motility, photoactivation in PI(3)K inhibited cells could induce protrusions but not motility. Additionally, Rac activation could not induce proper F-actin polarization in PI(3)K-inhibited cells. This suggested a two-tiered model of PI(3)K activity in migrating neutrophils, where PI(3)K was needed for Rac-mediated leading edge protrusion but was also necessary for Rac-independent F-actin polarization [40].

The question of how neutrophils are recruited to wounds has also been partially answered by the application of advanced imaging techniques to zebrafish research. The fluorescent, reversible, genetically encoded, ratiometric probe, HyPer, is able to detect hydrogen peroxide production in vivo [44]. Niethammer et al. used this probe to show that wounding zebrafish fins induced a burst of hydrogen peroxide that was necessary for the early recruitment of neutrophils to wounds [45]. Wound-produced hydrogen peroxide was subsequently demonstrated to attract neutrophils through the oxidation-mediated activation of the src-family kinase, Lyn. The ability to perform a neutrophil-specific rescue with wild-type and oxidation-mutant Lyn, a major benefit of the zebrafish system, was critical to confirming this finding [46]. Overall, these advances have demonstrated the value of coupling fluorescent bioprobes and fluorescent-tagged proteins to live, in vivo studies of neutrophil behavior. The continued innovation of advanced imaging techniques will be critical to future advances in understanding neutrophil behavior.

## 4. Neutrophils Leave Wounds via Reverse Migration

 Prior to the observation of neutrophil reverse migration, the previous paradigm of neutrophil responses, based on mammalian studies, was that neutrophils underwent apoptosis after responding to an inflammatory stimulus [5, 6]. The process of macrophage clearance of apoptotic neutrophils in tissues has been well established [4, 7]. However, a previous study using an experimental rat model of nephritis showed that intravascular neutrophils do not necessarily undergo apoptosis but can leave a site of inflammation through glomerular capillaries, suggesting that alternative mechanisms may mediate resolution of neutrophil-mediated inflammation [47]. In support of this idea, in vivo imaging of zebrafish neutrophils was the first direct demonstration that reverse migration was responsible for clearance of neutrophils from the interstitium of wounded tissues [8]. Indeed, studies of neutrophil reverse migration in zebrafish larvae have found that neutrophil apoptosis at a wound site is a rare event, occurring in less than 3% of the responding neutrophils [48]. Mathias et al. were able to demonstrate this reverse migratory behavior by tracking wound-responsive neutrophils in the transgenic (Tg) zebrafish line, Tg(mpx : GFP), in which GFP is expressed specifically in neutrophils. This study further demonstrated that neutrophils undergoing both forward and reverse migration to a wound had nearly equivalent velocity and directionality, implying that each was a robust, active process [8]. Using zebrafish, other groups have also observed neutrophil reverse migration under similar experimental conditions [38, 48, 49].

**Figure 1:**
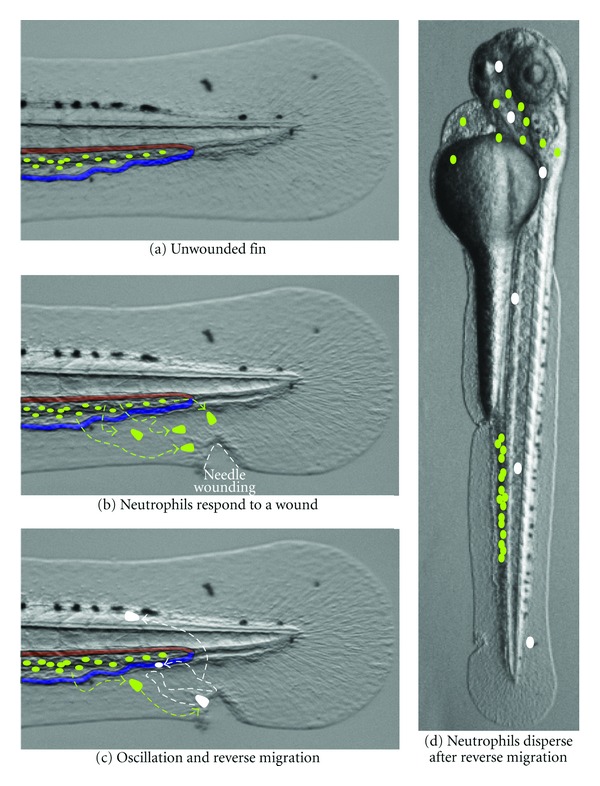
Neutrophil reverse migration in zebrafish larvae. This diagram illustrates the behavior of neutrophils undergoing reverse migration over an image of a 3-day postfertilization zebrafish larva that was PTU-treated to prevent pigmentation. (a) In unwounded larva, neutrophils (green ovals) are present in the caudal hematopoietic tissue (CHT), which is situated between the caudal artery (red shading) and the caudal vein (blue shading). (b) In response to a wound, neutrophils are mobilized from the CHT, and they migrate through the tissue towards a wound. (c) The green to white color change represents the ability to photoconvert individual neutrophils that reach a wound. Neutrophils often migrate between the wound and the vasculature multiple times before finally departing. Neutrophils have been observed performing reverse migration by entering the vasculature and by migrating through tissues in zebrafish larvae. (d) Reverse migrated neutrophils (white) are found dispersed throughout the body without an obvious tissue preference 2 days after leaving wounds.

 While illuminating, technical challenges prevented the exploration of some questions about the fate and individual behavior of neutrophils responding to a wound. The application of the photoconvertible protein, Dendra2, which can be switched from green to red fluorescence with 405 nm light, allowed these questions to be more definitively explored [50]. Dendra2-labeled neutrophils that had reached a wound were photoconverted, permitting detailed tracking of a small number of neutrophils. Thus, it was determined that individual neutrophils often traffic between the wound and the vasculature repeatedly before leaving permanently. The significance of this oscillatory behavior remains unclear, but it may reflect the competition of signals between two endpoints. Transgenic zebrafish with GFP-labeled vasculature, Tg(fli1 : EGFP), demonstrated that some reverse-migrating neutrophils do enter the vasculature, performing a true reverse transendothelial migration (Figures 1(a)–1(c)). While the oscillatory neutrophil migration and reverse transendothelial migration appear to be steps along a common pathway in the movement of neutrophils away from inflamed tissue, it is not yet clear what triggers the progression between these steps. Finally, the fate of neutrophils that responded to wounds was determined by tracking photoconverted neutrophils for two days after wounding, demonstrating two important findings: neutrophils survived for multiple days after wounding, and they were found dispersed throughout the body without obvious tissue preferences (Figure 1(d)) [9]. While these observations of neutrophil reverse migration in zebrafish were intriguing, they still faced criticism that this could be a zebrafish or larva-specific phenomenon and not applicable to mammals.

 Around the time of the first observation of neutrophil reverse migration in zebrafish, two groups made observations that suggested the existence of neutrophil reverse migration in mammalian systems. Primary human neutrophils that were cocultured on monolayers of endothelial cells in vitro were observed to transmigrate through the endothelium and subsequently perform reverse transendothelial migration to return to the apical surface [51]. These reverse transmigrated (RT) neutrophils demonstrated decreased adhesion to the endothelial surface and decreased tendency to undergo forward transendothelial migration as compared to “fresh” neutrophils. Another interesting finding from this study was a unique surface phenotype for neutrophils that had undergone the reverse migration process. CXCR1, the receptor for the neutrophil chemoattractant IL-8, CD11b (integrin *α*_M_ chain), and CD54 (intercellular adhesion molecule-1) along with other cell surface markers were used to distinguish the different neutrophil populations. RT neutrophils were found to be CD11b^high^CD54^high^CXCR1^low^, which differentiated them from freshly isolated (CD11b^low^CD54^low^CXCR1^high^) neutrophils [51]. Additionally, a study by Maletto et al. found that neutrophils in mice that had been immunized against ova peptide would transport an FITC-labeled ova peptide from the site of footpad injection to local lymph nodes. Extensive flow cytometric and histopathologic analysis demonstrated that the cells bearing ova-FITC were indeed neutrophils. One caveat of this study was that ova-FITC containing neutrophils were only found in lymph nodes in the ipsilateral, but not contralateral leg, to the site of ova administration, implying that lymphatic drainage may have been responsible for their dissemination [52]. While not definitive proof of reverse migration, these findings supported the idea that neutrophils could survive after responding to an inflammatory stimulus and could affect the immune response in a manner spatiotemporally separated from the site of this stimulus. Subsequent in vivo studies in mice have also demonstrated that neutrophils can directly interact with B cells and T cells in lymphoid tissue, further strengthening the concept of neutrophils existing outside of their conventional roles [10, 53–55].

 The most direct support for the observations of reverse migration in zebrafish has come from a recent intravital imaging study using mice. Woodfin et al. used a system in which intrascrotal inflammation, induced by ischemia-reperfusion injury, allowed the monitoring of fluorescently-labeled neutrophils. Approximately 10% of the transendothelial migration events observed with this assay were reverse transendothelial migration. Additionally, it was observed that ischemia-reperfusion injury disrupted the localization of junctional adhesion molecule C (JAM-C) to endothelial junctions and that mice with JAM-C^−/−^ endothelial cells demonstrated an increase in reverse transendothelial migration, reaching greater than 50% of total transendothelial migration events [10]. It is important to note that this study did not address the migration of neutrophils in the tissue parenchyma, and this will be an interesting area for future study. These findings of neutrophil reverse migration in mice and in vitro are paralleled by monocyte studies that demonstrated reverse transendothelial migration with similar kinetics and regulation by JAM-C [56, 57]. This suggests a possible conservation in the mechanisms that mediate reverse transendothelial migration of neutrophils and macrophages.

 Several differences have been observed between zebrafish and mammalian reverse migration that await further investigation. While all of the reverse migration events described thus far in mice involve transendothelial migration, some neutrophils are able to disperse throughout the body of the zebrafish larva without entering the vasculature. Most apparent is that nearly all wound responsive neutrophils perform reverse migration in zebrafish, whereas approximately 10% do so under the observed conditions in mice [9, 10]. We believe that this discrepancy may be the result of using larva versus adult animals, species-specific differences, or the type of inflammatory stimulus. However, the high percentage of reverse migrating neutrophils may provide a substantial benefit in studies of reverse migration. Mammalian studies have supported the utility of using zebrafish to study reverse migration; however, neither system has yielded a definitive answer on the mechanisms that mediate reverse migration.

## 5. The Mechanisms Driving Reverse Migration

Recent work in zebrafish has implicated hypoxia-inducible factor-1*α* (Hif-1*α*) in neutrophil reverse migration. Pharmacologic stabilization of Hif-1*α* or the expression of a dominant active Hif-1*α* impaired resolution of inflammation by neutrophil reverse migration [48]. While this is a promising first step, it does not appear that Hif-1*α* is the dominant factor regulating reverse migration.

The observed behavior of reverse migrating neutrophils, as described above, can allow us to speculate on the potential signaling mechanisms that are relevant to this process. As reverse migration occurs both during active inflammation and as part of the local resolution of neutrophil-mediated inflammation, there may be two temporally distinct phases of this process. During the early response to a wound, velocity and directionality are equivalent during forward and reverse neutrophil migration, suggesting that reverse migration is an active process [8]. Therefore, a passive mechanism, such as the loss of wound-derived chemoattractants and the random dispersal of neutrophils, is not likely to be involved.

**Figure 2:**
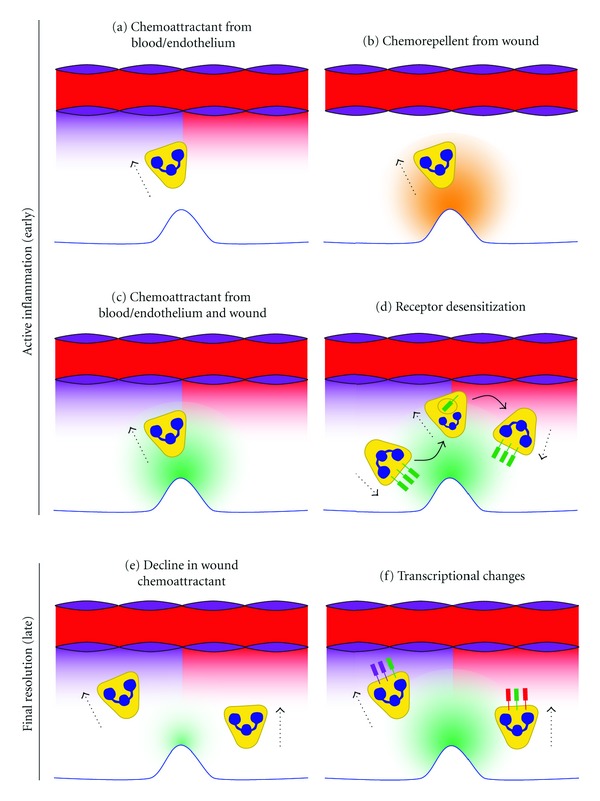
Proposed mechanisms for reversed migration. This diagram illustrates how chemoattractant gradients from the blood (red), endothelium (purple), and wound (green) or chemorepellent gradients (orange) may influence reverse migration. The color of a chemoattractant receptor matches the gradient to which it responds. (a)–(d) Reverse migration in the early wound response. (a) Demonstration of neutrophil reverse migration towards blood or endothelium-derived chemoattractants. (b) Reverse migration of a neutrophil away from a wound-derived chemorepellent. There could be competition between wound-derived chemoattractants (not shown) and chemorepellents promoting reverse migration, or neutrophils may perform fugetaxis from areas of high chemoattractant concentration. (c) Oscillatory behavior of neutrophils suggests competing gradients of chemoattractants may exist between the wound and vasculature. (d) Receptor desensitization, via internalization or other mechanisms, may allow neutrophils to oscillate between the wound and the vasculature while others are still actively responding to the wound. (e)–(f) Mechanisms that promote resolution of neutrophil-mediated inflammation at wounds. (e) During the healing phase, wounded tissue may gradually produce less neutrophil chemoattractant, shifting the balance to favor reverse migration. (f) Neutrophils that responded to a wound may initiate transcriptional changes favoring reverse migration from the wound. Potential changes include altered expression or sensitivity of chemoattractant receptors.

We speculate that the signals that trigger neutrophils to perform reverse migration could include a competing chemoattractant “pulling” them away from the wound, a chemorepellent “pushing” them away from the would, or both (Figures 2(a) and 2(b)). Because neutrophils often migrate back to the vasculature after an inflammatory response, chemoattractants emanating from the blood or endothelium are attractive targets for promoting migration away from a wound (Figure 2(a)). Interestingly, high concentrations of chemoattractants, including IL-8 (CXCL8), can repel neutrophils in vitro and in vivo [58]. Other leukocytes can also be repelled by high chemokine concentrations. T cells are repelled by high concentrations of SDF-1 (CXCL12) in vivo and in vitro [59], and monocytes can be repelled by high concentrations of eotaxin-3 (CCL26) [60]. Thus, it is also plausible that the wounded tissue could be a source of both chemoattractants and chemorepellents in competition with each other. Previous studies of leukocyte chemorepulsion suggest that a wound chemoattractant at sufficiently high concentration could also act as a chemorepellent. As a neutrophil approached the wounded tissue, the concentration of chemorepellent would increase, potentially overwhelming the effect of the chemoattractant and driving the neutrophil away from the wound (Figure 2(b)).

Neutrophils respond to chemokines in a hierarchical manner, preferring some over others, when faced with competing gradients [61, 62]. The oscillatory migration of neutrophils between wounds and the vasculature suggests that a mechanism of competing chemoattractant gradients between these locations is likely (Figure 2(c)) [8, 9]. In this scenario, the signals promoting migration away from a wound (Figures 2(a)-2(b)) would compete with the signals attracting neutrophils to wounds. One complicating factor for this model is that “fresh” neutrophils continue to arrive at wounds after some have already reverse migrated, suggesting that neutrophil intrinsic regulation may also be involved in their oscillatory behavior and eventual departure. Therefore, we believe that the most likely explanation for the oscillatory behavior during the early wound response is a combination of competing chemokine gradients and neutrophil-autonomous changes in chemokine receptor sensitivity. It is known that neutrophils will internalize and downregulate G protein coupled receptors, including many chemokine receptors, after stimulation [63]. In this model, as neutrophils approach a high concentration of chemoattractant at the wound or vasculature, receptor desensitization would occur and promote migration towards the competing gradient (Figure 2(d)). Failure to internalize the CXCR4 receptor prevents downregulation of CXCR4-mediated signalling and is responsible for the retention of neutrophils in the bone marrow or caudal hematopoietic tissue of WHIM syndrome patients and a zebrafish model of WHIM syndrome, respectively [24, 64–67]. This suggests that the dynamic regulation of chemokine receptor surface expression or its sensitivity to signaling is critical for allowing neutrophils to follow the appropriate gradient of chemoattractant, which could be necessary for performing reverse migration.

The eventual permanent departure of neutrophils from the wound site indicates the second phase of the reverse migration response. During this later phase, the wounded tissue may gradually produce less chemoattractant or more chemorepellent as it heals, shifting the balance towards reverse migration (Figure 2(e)). The time it takes for neutrophils to leave a wound, which can be several hours, makes it possible that transcriptional changes may also be involved. In a mechanism that may be cooperative with declining chemoattractant gradients, neutrophils could be programmed to activate transcriptional changes that favor reverse migration after responding to an inflammatory stimulus. The result could modify chemokine receptor expression or sensitivity, shifting the balance towards reverse migration (Figure 2(f)). Clearly, there are many possible mechanisms internal and external to the neutrophil that may drive reverse migration, making this an area ripe for rapid advancement.

## 6. Potential Roles for Reverse Migrated Neutrophils

 While current studies have not demonstrated a definite role for reverse migrated neutrophils, the recent findings of several groups have challenged the idea that neutrophils are short-lived cells with narrowly defined functions. Because reported neutrophil half-lives were less than 12 hours and there was no knowledge of neutrophil reverse migration, neutrophils were not thought to have immunomodulatory roles other than through the cytokines and effectors that they produced at sites of inflammation. However, recent reports have challenged the short lifespan of neutrophils. Although controversial, a recent study used ^2^H_2_O labeling to determine that the in vivo half-life of human neutrophils was 3.8 days (total lifespan: approximately 5.4 days) [68–71]. Others have also reported neutrophil lifetimes that were longer than 24 hours. Neutrophils that underwent reverse transendothelial migration, trafficked to lymph nodes, or were cocultured with TNF-*α*/IL-17 stimulated synovial fibroblasts had their expected lifetimes extended [51, 52, 72]. Zebrafish neutrophils that underwent reverse migration could also be found for at least 2 days after they had left a wound [9]. While evidence supports the existence of reverse migration and prolonged neutrophil life in vivo, data supporting either a proinflammatory or anti-inflammatory role for reverse migrated neutrophils remain plausible.

**Figure 3:**
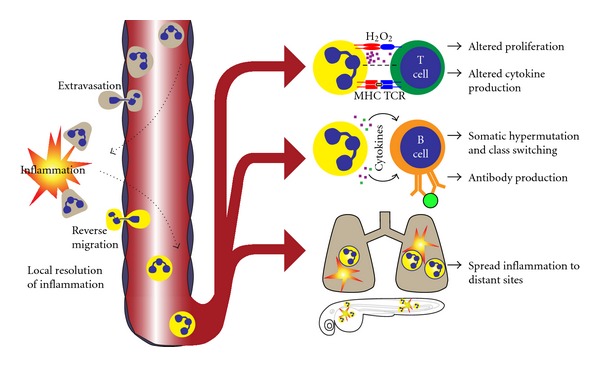
Potential functions of reverse migrated neutrophils. On the left side of the illustration, neutrophils (tan) are responding to an extravascular inflammatory stimulus. After responding to the stimulus, neutrophils perform reverse migration (yellow) and enter the vasculature. This process has been suggested as a mechanism to resolve inflammation at the local level. We are proposing the following as potential functions of reverse migrated neutrophils. Neutrophils may modulate T-cell proliferation and cytokine production in an antigen-independent or-dependent manner. Integrin-mediated neutrophil-T cell contact, hydrogen peroxide, and T cell receptor (TCR) signaling have demonstrated importance in neutrophil-mediated regulation of T cell function. Neutrophils may promote antibody diversification, class switching, and production by splenic B cells through the secretion of cytokines. Reverse migrated neutrophils may travel to distant tissues and induce additional inflammation. Reverse-migrated neutrophils were implicated in inducing pulmonary inflammation in mice.

 An intriguing correlation between studies of reverse migrated neutrophils and immunomodulatory neutrophils is that these populations appear to have an activated phenotype that is characterized by elevated CD11b and elevated CD54 expression (Table 1) [10, 51–55]. CD11b^high^ neutrophils were found to transport fluorescent antigen to lymph nodes and survive for an extended period. Although direct interaction with lymphocytes was not documented, neutrophil depletion resulted in increased IL-5 production, which suggested that neutrophils could be altering cytokine production by CD4^+^ T cells [52]. A more recent study found that CD11b^high^CD54^high^CD62L^low^CD16^high^ neutrophils were induced after injecting healthy human subjects with LPS or in severely injured trauma patients, and that this neutrophil population was capable of inhibiting antigen-dependent and- independent T cell proliferation. Catalase treatment or Mac-1 integrin (CD11b/CD18) blocking decreased the neutrophil inhibitory function, and imaging revealed that H_2_O_2_ was produced at neutrophil T cell contacts, suggesting a model in which activated neutrophils could form a synapse-like structure and deliver proliferation-inhibiting H_2_O_2_ to T cells (Figure 3) [54]. Another interesting population of CD11b^high^CD54^high^ neutrophils was recently found in the marginal zone of the spleen. These neutrophils expressed MHC class II, which is normally found on professional antigen presenting cells and had the ability to promote antibody diversification and production by splenic B cells. These neutrophils populated the splenic lymphoid follicles after acquiring gut-associated-microbial products, suppressed T cell proliferation, and promoted antibody production to T cell-independent antigens (Figure 3) [55]. Taken together, these findings support the idea that neutrophils are able to acquire material from extravascular tissue, return to and survive in lymphoid tissue, and modulate the adaptive immune response. The ability to retrieve antigen outside of the bloodstream and the surface phenotype consistent with reverse migrated neutrophils suggests the possibility that neutrophils could perform reverse migration during their trip back to lymphoid tissues.

 While the effects of neutrophils on the adaptive immune system described above could be viewed as anti-inflammatory or immunomodulatory, a proinflammatory role has also been proposed for reverse migrated neutrophils [9, 10, 51]. Several lines of evidence lend support to this hypothesis, which stems from the observations that severe, localized trauma can lead to multiple organ failure and neutrophil reverse migration. Neutrophils are thought to be important in the pathogenesis of multiple organ failure, which is associated with states of injury and heightened inflammation [73, 74]. While the reverse migration of neutrophils away from a site of inflammation may result in local resolution of inflammation, the observation by Yoo and Huttenlocher that reverse migrated neutrophils disperse in tissues throughout the body of zebrafish suggests the possibility that neutrophils could be promoting inflammation or tissue damage in these sites (Figure 3) [9]. Additionally, proinflammatory conditions such as ischemia-reperfusion injury, rheumatoid arthritis, and chronic colitis generate elevated numbers of neutrophils with the reverse-migrated surface phenotype [10, 51, 53]. Mice with chronic colitis were found to have neutrophils that presented antigen to T cells in an MHC II-restricted manner, resulting in increased T cell proliferation and proinflammatory cytokine production [53]. Furthermore, after ischemia-reperfusion injury in mice, pulmonary edema was observed, and neutrophils with a reverse migrated surface phenotype could be found in the lung, suggesting that this neutrophil population may promote inflammation at sites distant from the actual injury (Figure 3) [10]. Reverse migrated neutrophils also produce elevated amounts of reactive oxygen species, which are thought to play a key role in the pathophysiology of multiple organ failure [10, 51, 73, 74]. Although a pro- or anti-inflammatory role for reverse migrated neutrophils remains uncertain, many lines of evidence support the idea that neutrophils can retrieve antigen from extravascular tissues, move to distant organs, and influence the adaptive immune response, leading us to believe that neutrophil reverse migration could be playing a role in these neutrophil functions.

## 7. Conclusions and Future Directions

 The last five years have yielded exciting developments in the study of neutrophil biology, including the process of reverse migration, which are rapidly changing the view that neutrophils are short-lived cells with narrowly defined effector functions. It seems that in at least some circumstances neutrophils can regulate T cell activity and present antigen in the context of MHC II, functions which were previously ascribed to macrophages and dendritic cells, the professional antigen presenting cells [52–55]. While evidence in support of the existence of reverse migration in mammals continues to grow, the mechanisms driving reverse migration and the functions of reverse migrated neutrophils remain to be further defined.

 Continued progress towards these goals will require additional characterization of neutrophil forward and reverse migration coupled with technical advances. In order to fully understand the signaling that drives reverse migration, we must better characterize the signaling that is recruiting neutrophils to wounds. Currently, we know that a gradient of H_2_O_2_ drives the early recruitment of neutrophils to wounds. However, neutrophils still arrive at wounds with a 30–60 minute delay in the absence of wound-produced H_2_O_2_, indicating that other chemoattractants are likely involved at later time points [46]. Additionally, the chemoattractants or chemorepellents that drive neutrophil reverse migration remain entirely unknown. The intracellular signaling that differentiates forward from reverse migration is also unexplored. Fully characterizing this signaling hierarchy would be facilitated by chemical or genetic screening strategies with the ability to read out changes in neutrophil-mediated inflammation.

 Advances in our imaging capabilities in zebrafish will also be critical to further progress in reverse migration studies. While we can observe the movements of neutrophils responding to wounds in zebrafish, we currently know little about their other functions in vivo. Tools that allow live imaging of neutrophil activation and effector functions—reactive oxygen species production, phagocytosis, and degranulation—will be particularly valuable. Biosensors that allow the activity of critical signaling pathways to be monitored will help to integrate the roles of extracellular cues and neutrophil effector functions on their wound responses. A FRET-based Rac activity biosensor, which has been applied to the study of primordial germ cell protrusion and migration, is an example of the type of probe that will be integral in understanding the signaling pathways controlling reverse migration [75, 76].

 Determining the function of reverse migrated neutrophils should also be a priority. Recent reports that neutrophils can modulate B cell and T cell functions demonstrate the importance of characterizing these interactions in vivo. Approaching this question with 2–4-day-old zebrafish larvae is not possible, as they have not yet developed an adaptive immune system [16]. As a result, the functions of zebrafish B and T cells in response to inflammatory stimuli are poorly characterized. Techniques that allow simultaneous in vivo imaging of neutrophils, B cells, T cells, and effector molecules in more developed zebrafish would allow a more definitive determination of how these interactions shape immune responses.

 In order to fully understand the role of neutrophil reverse migration, it will be necessary to determine how it impacts immune homeostasis and disease. Models of immunodeficiency and inflammatory disease have been developed in zebrafish larvae [22, 23, 77, 78]. However, a model of neutrophil autonomous inflammatory disease has not yet been developed in zebrafish. These and future disease models can be used to determine if neutrophil reverse migration is altered in pathologic states. Additionally, determination of the signaling that drives reverse migration will allow this process to be inhibited, which will be informative in understanding how it may influence pathology.

While rapid progress has been made in the characterization of reverse migration in zebrafish and mice, much remains to be learned about the underlying mechanisms and functional consequences of this process. However, the availability of powerful tools for genetic manipulation and in vivo imaging makes it clear that the use of transparent zebrafish larvae will allow researchers to continue probing the secrets of neutrophil behavior in vivo.

## Figures and Tables

**Table 1:** Comparison of surface phenotypes between studies of neutrophil reverse migration and immunomodulation by neutrophils. CD11b (integrin *α*_*M*_), which is a component of Mac-1 integrin, and CD54 (ICAM-1) were the surface molecules with the most overlap between these studies. Blank spaces indicate that the expression of this molecule was not addressed by a particular study. The (↑) indicates that expression of the indicated molecule was elevated over the appropriate control sample of neutrophils (non reverse migrated, not responsible for neutrophil effects on lymphocytes, etc.).

	CD11b	CD54
Reverse migration		
Buckley et al.2006 [51]	↑	↑
Woodfin et al.2011 [10]		↑
Immunomodulation		
Maletto et al.2006 [52]	↑	
Ostanin et al.2012 [53]	↑	↑
Pillay et al.2012 [54]	↑	↑
Puga et al.2012. [55]	↑	↑
